# Intersectional Approaches to Minority Aging Research

**DOI:** 10.1007/s40471-022-00317-5

**Published:** 2023-01-10

**Authors:** Courtney S. Thomas Tobin, Ángela Gutiérrez, Heather R. Farmer, Christy L. Erving, Taylor W. Hargrove

**Affiliations:** 1 Department of Community Health Sciences, Fielding School of Public Health, University of California Los Angeles, Los Angeles, CA USA; 2 Department of Social Medicine, Heritage College of Osteopathic Medicine, Ohio University, Irvine 128B 57 West Oxbow Trail, 1 Ohio University Drive, Athens, OH 45701-2979 USA; 3 Department of Human Development and Family Sciences, University of Delaware, Newark, DE USA; 4 The University of Texas at Austin, Austin, TX USA; 5 Department of Sociology, University of North Carolina at Chapel Hill, Chapel Hill, NC USA

**Keywords:** Intersectionality, Minority aging, Minoritized populations, Black Americans, Latinx

## Abstract

**Purpose of Review:**

Growing racial/ethnic diversity among America’s older adults necessitates additional research specifically focused on health and well-being among aging minoritized populations. Although Black and Latinx adults in the USA tend to face worse health outcomes as they age, substantial evidence points to unexpected health patterns (e.g., the race paradox in mental health, the Latino health paradox) that challenge our understanding of health and aging among these populations. In this review, we demonstrate the value of intersectionality theory for clarifying these health patterns and highlight the ways that intersectionality has been applied to minority aging research. To advance the field, we also make several recommendations for incorporating intersectional approaches in future scholarship on minority aging.

**Recent Findings:**

Scholars have applied intersectional approaches to health and aging to unravel how social statuses and social conditions, such as race, ethnicity, gender, nativity, incarceration history, geographic region, and age, produce distinct shared experiences that shape health trajectories through multiple mechanisms.

**Summary:**

We highlight common intersectional approaches used in minority aging research and underscore the value of this perspective for elucidating the complex, and often unexpected, health patterns of aging minoritized populations. We identify several key lessons and propose recommendations to advance scholarship on minority aging.

## Intersectional Approaches to Minority Aging Research

Growing racial/ethnic diversity among America’s older adults [1] necessitates research focused on health and well-being among aging minoritized populations. Although Black and Latinx adults in the USA tend to face worse health outcomes as they age [2, 3], substantial evidence points to unexpected health patterns that challenge our understanding of health and aging among these populations. For example, rates of major psychiatric disorders (e.g., major depression) are relatively low among Black Americans despite elevated levels of non-specific psychological distress (e.g., depressive symptoms), heightened prevalence of chronic physical health conditions (e.g., hypertension, diabetes, heart diseases), early onset of physical disability, and high rates of premature mortality across the life course [4, 5]. Often referred to as “the race paradox in mental health [5–8],” this set of research findings is counterintuitive because many physical and mental health conditions have shared etiologies and are shaped by similar risk factors in the general population [4, 6–8]. Moreover, Black Americans collectively face greater life course exposure to contextual and psychosocial health risks such as social and political marginalization [9], neighborhood and individual-level socioeconomic disadvantage [10–12], and heightened exposure to social stressors (e.g., discrimination) [13, 14], all of which have been linked to adverse physical and mental health outcomes. Though studies have assessed potential explanatory mechanisms for the paradoxical health trends of Black Americans, including measurement error (i.e., Black and White individuals respond differently to mental health assessments) [7], coping [15, 16], and prescription drug usage [17], such patterns remain poorly understood.

Prior research also documents a “Latino Health Paradox,” referring to the favorable health of Latinx adults relative to non-Latinx Whites. Specifically, the Latinx population fares better than non-Latinx Whites on several indicators of health, such as mortality, longevity, and cardiovascular disease [18–21], despite Latinx adults having lower average socioeconomic status (SES) [22], lower likelihood of having health insurance [23], and greater exposure to psychosocial and physical risk factors [24, 25] than their White counterparts. However, accumulating evidence suggests that their longer life expectancies, often referred to as the “Latino mortality advantage,” do not shield the Latinx population from other physical, mental, and cognitive health disadvantages and disparities [26–28]. Some scholars argue that cultural values may confer resilience to this group by promoting social support and social capital across distinct levels (e.g., intrapersonal) and contexts (e.g., family, community) [29, 30]. Other explanations focus on the role of immigration. For example, the “healthy immigrant hypothesis” suggests that Latinx individuals who migrate to the USA are positively selected on health; however, their health tends to erode with more time spent in the USA given the stressors of acculturation. Additionally, the “salmon bias hypothesis” posits that Latinx individuals who become sick return to their country of birth, leading to a residual of “healthy” Latinx individuals in the USA [31]. While prior work has provided some support for these hypotheses, the processes linking immigrant status, ethnicity, and health are complex, and more research is needed to identify factors that promote well-being and reduce comorbidities among the aging Latinx population.

Collectively, these unexpected epidemiological phenomena continue to puzzle scholars, as they challenge our understanding of the social determinants of health and aging among minoritized populations. In this review, we demonstrate the value of intersectionality theory for clarifying these health patterns and highlight the ways that intersectionality has been applied to minority aging research in the last few decades. We also make several recommendations for incorporating intersectional approaches in future scholarship on minority aging.

### Intersectionality Theory

To better understand the health trajectories of aging minoritized populations, scholars have increasingly advocated for and utilized intersectional perspectives. Rooted in Black feminist epistemology [32–34], the earliest iterations of intersectionality theory aimed to highlight how Black women’s “doubly disadvantaged” race and gender statuses made them especially vulnerable to gendered violence and capitalist exploitation [35]. Later, Collins [34] emphasized the “matrix of domination” in which systems of race, class, gender, sexuality, and ability oppression (among others) intersect to locate and either constrain or enable individuals based on their multiple intersecting statuses and social identities [35]. As such, intersectionality theory considers how social structural dynamics and systems of oppression such as racism, sexism, capitalism, and heteropatriarchy intersect and reinforce each other to stratify and dominate minoritized groups by shaping the contexts of their lives [2, 35–37].

### Health, Aging, and Intersectionality

Though health and aging processes are produced by overlapping and interlocking systems of privilege and oppression [38], many studies do not account for these complex mechanisms. Nevertheless, scholars have increasingly advocated for the use of intersectionality as a critical framework within aging research [39, 40]. An intersectionality approach to health and aging recognizes that statuses like race, ethnicity, gender, and age are fundamental determinants of opportunity whose effects on health cannot be truly disaggregated or understood independently because they simultaneously define exposure to health risks as well as access to health-promoting resources [2, 41]. With this lens, the aging process involves more than just the physical changes associated with getting older or the cumulative impact of other inequities over the life course. Rather, age is a source of inequities in its own right (e.g., ageism), and an intersectional approach to aging theorizes how people experience such bodily changes over time [40].

Intersectionality theory may be especially critical for understanding the health and aging of minoritized populations, who face challenges associated with being both older and members of minoritized racial groups [42]. Although social gerontologists often mistakenly equate intersectionality with attention to diversity or differences [40], an intersectional approach intends to move beyond the observation of group differences to specify how groups are related in terms of the structural roots of their experiences of marginalization [37]. Individuals’ social positionality is based on multiple social statuses (e.g., race, ethnicity, age, gender) that produce distinct shared experiences that shape health trajectories through multiple mechanisms [36]. Thus, by utilizing intersectional approaches to investigate health and well-being among minoritized aging populations, we become better positioned to recognize the structural processes that produce health inequalities and to identify the unique combinations of health-relevant social conditions and contexts they experience [43, 44]. Failure to incorporate these approaches may obscure the social processes underlying disparities across the life course.

### Intersectionality in Minority Aging Research

Given the broad array of minority aging research, there has been great diversity in the application of intersectionality theory to this area. Studies have shown that intersectional approaches to minority aging reveal considerable heterogeneity in various indicators of health across race, ethnicity, gender, SES, nativity, and age, and these approaches have varied over time. Some of the earliest examples of intersectionality theory applied to issues of minority aging used “configurational approaches” [45, 46], which involves comparisons between status groups. In such cases, a study might compare race-gender categories to a single reference group, usually White men [2, 46, 47]. While insightful, this method often assumes an additive relationship between statuses, implying that they operate independently of one another and that disadvantaged social positions work in equivalent ways to shape health risks and resources. Scholars have criticized such additive explanatory models, however, arguing that they erroneously assume that disadvantages incurred by Black and Latina women are the sum of those associated with being female and those associated with being a person of color [46]. Others assert that additive approaches are insufficient for capturing the realities of diverse aging groups because they ignore the full extent and interdependence of systems of inequality [48, 49].

Instead, intersectionality theory is more appropriately applied with the consideration of the multiplicative relationships among statuses. Multiplicative approaches recognize that social statuses are interdependent and often combine multiplicatively to mutually construct the experience and consequences of one another [48, 50, 51]. Hence, this approach emphasizes that individuals located at similar positions in the social hierarchy (e.g., Black women) may have shared, but not equivalent experiences [32]. A growing number of minority aging studies have incorporated multiplicative approaches, testing the interactive consequences of social statuses. For example, Ailshire and House [52] documented social disparities in body mass index (BMI) at the intersection of race, gender, SES, and age. By estimating interactions among these statuses, the researchers found that low SES Black women experienced the greatest increases in BMI with time, while high SES White men experienced the least amount of BMI growth. Interestingly, these differences by race, gender, and SES were largely observed among young adults and diminished with age. Later, Brown and colleagues [53] integrated intersectionality theory with life course perspectives to evaluate whether the joint health consequences of racial/ethnic, gender, and socioeconomic stratification were additive or multiplicative and whether such consequences varied over time among White, Black, and Latinx older adults. Evidence from this study indicated that the consequences of social statuses were indeed interactive, resulting in the greatest racial/ethnic inequalities in health among women and those with higher levels of SES, although inequalities tended to decline with age. More recently, Farmer, Wray, and Haas [54] clarified the associations among race, gender, SES, and C-reactive protein (CRP), an indicator of chronic inflammation, among older Black and White adults. Results from the study indicated that Black women with less than a high school education had the highest CRP of all groups in the sample. Moreover, while an expected educational gradient in CRP was observed among White adults, there was a less consistent relationship between education and CRP among Black adults, particularly Black men. In a similar manner, Hamler and colleagues [55] documented a distinct, gendered influence of COVID-19-related stressors on the psychological well-being of Black and White older adults, finding that the association between COVID-19-related stressors and psychological distress varied by race among men only.

While most intersectional studies have focused on social statuses such as race/ethnicity, gender, and SES, minority aging researchers have increasingly evaluated the combined significance and nuances of other statuses and social identities. For instance, some studies have provided additional insight into the ways that self-identified race combines with other identities. López and colleagues [56] considered the multidimensionality of race among Latinx adults by distinguishing between “street race” (how individuals believe they are classified by others in the USA), self-perceived race (how individuals self-classify), and socially assigned race. They examined how these multiple dimensions of race interacted with gender to shape mental and physical health among this population. Among Black Americans, scholars have demonstrated that skin color represents an added dimension of social inequality that intersects with statuses like age and gender to shape health across the life course. For example, Hargrove [57] assessed the ways that skin color and gender interacted to shape body weight trajectories among middle-aged and older Black adults, documenting particularly disadvantaged BMI trajectories among dark-skinned Black women and relatively advantaged BMI trajectories among medium-skinned Black men. More recently, Hamler and colleagues [58] found that skin color significantly modified the association between discrimination and mental health among older Black Americans. Specifically, the association between discrimination and mental health was stronger among darker skinned respondents than their lighter skinned counterparts, particularly among Black men. Taken together, these studies demonstrate the added value of an intersectional approach when evaluating the health significance of race and race-related dimensions for aging minoritized populations.

Researchers have also explored the ways that ethnicity and nativity intersect with other identities to shape health trajectories among minoritized populations. For instance, Brown [3] integrated intersectionality and life course perspectives to evaluate interactions between race/ethnicity, nativity, and age, finding that US- and foreign-born Black and Latinx older adults not only experience earlier health deterioration than US-born White Americans, but that they also tend to exhibit steeper health declines with age. The study also challenges monolithic assumptions about healthy immigrant and erosion processes because these processes are contingent on both race/ethnicity and age. That is, while White immigrants have a persistent health advantage, Black and Latinx immigrants experience a health disadvantage that increases with age. Garcia and colleagues [59] documented additional nuances among Latinx subpopulations, such that older US-born Latinx adults had poorer cognitive function than non-Latinx Whites and foreign-born Latinxs. Investigating the relationship between multiple chronic conditions (MCC) (e.g., heart disease, cancer) and depressive symptoms, Erving and Frazier [60] also identified race-gender-nativity nuances among Black, Latinx, and White older adults; specifically, MCC was the most distress-inducing for foreign-born Latina women, a socially marginalized group who experiences oppression attributable to structural disadvantage at the intersection of race, nativity, and gender. Moreover, though MCC rates were highest among Black women, they did not appear to psychologically succumb to its detrimental effects. Such findings may begin to unravel the complex ways in which physical and mental health patterns are nuanced by the intersecting social statuses of race, ethnicity, nativity, and gender.

While studies examining the roles of ethnicity and nativity are more common among older Latinx adults, there has also been growing interest in the ways that these processes influence health among older Black Americans. For instance, in a study examining the association between stress exposure and physical health among older Black women, Erving [61] found that older Caribbean Black women experienced better self-rated health, lower rates of multiple chronic conditions, and fewer functional limitations compared to their African American female counterparts. Moreover, the stressors associated with physical health varied by the ethnic identities of older Black women. When examining mental health patterns among older Black men, Mays and colleagues [62] found distinct patterns by ethnicity and nativity status. Counter to “healthy immigrant” expectations set forth in the broader health literature, foreign-born Caribbean Black men had higher risk for PTSD, depression, and alcohol abuse relative to their US-born Black male counterparts. This pattern of findings for physical and mental health among Black women and men reveals health heterogeneity within the older Black population that much of the gerontological literature has ignored [63].

Interestingly, minority aging scholars have also used intersectionality theory to consider how social conditions and contexts may interact with social statuses to shape health. Earlier this year, Latham-Mintus, Deck, and Nelson [64] demonstrated that the impact of incarceration history on health (i.e., physical limitations and depressive symptoms) varied by race/ethnicity and gender. Specifically, they observed that older women of color who were formerly incarcerated experienced the highest number of depressive symptoms and physical limitations compared to all other groups. Thierry [65] evaluated the interactive significance of race, neighborhood context, and geographic region for shaping telomere length (i.e., a maker of cellular aging) among older Black adults. This study revealed that older Black adults living in the Midwest who rated their communities more negatively had the shortest telomeres. Moreover, Hargrove and colleagues [66] examined the extent to which the relationship between education and cardiometabolic risk was varied by race/ethnicity, nativity, and the economic, policy, and social characteristics of early life counties of residence. Results suggested that educational disparities in health are robust for US-born Whites; for all other racial/ethnic-nativity groups, the health benefits of higher education depended on characteristics of the county in which young adults lived during childhood/adolescence. Such findings highlight important place-based factors that may contribute to complex health patterns by race, ethnicity, nativity, and education.

Collectively, this body of research highlights the numerous ways that intersectionality theory can shed new light on the health patterns of aging minoritized populations.

### Lessons Learned and Recommendations for Future Minority Aging Research

This review discussed some of the common intersectional approaches used in minority aging research and highlighted the value of this perspective for elucidating the complex, and often unexpected, health patterns of aging minoritized populations. As applications of intersectionality theory for minority aging research continue to evolve, however, it is important to recognize some of the valuable lessons learned from prior work and incorporate these strategies as we develop new strategies for future research. Below, we identify several key lessons and propose recommendations to advance scholarship on minority aging.

*First, intersectionality theory illuminates the structural and individual dynamics that produce health inequities across the life course, providing scholars with a framework with which to pinpoint important explanatory mechanisms shaping health and aging among minoritized populations.* As highlighted in Table 1, there are numerous theoretical approaches that may be applied to the study of minority aging. However, research on the health consequences of social stratification has typically focused on a single dimension of inequality at a time (e.g., race or gender), overlooking the simultaneous and interlocking impacts of multiple dimensions of inequality that are salient for the experiences of racially and ethnically minoritized people [38]. Intersectional approaches provide opportunities to overcome this issue by not only demonstrating the ways that social statuses, identities, contexts, and conditions intersect to produce varied health patterns [44], but it also allows us to identify the underlying mechanisms that explain those outcomes. Specifically, intersectionality provides a framework with which to study the specific factors that arise from intersecting social positions. For example, differences in factors such as exposure to social stressors (e.g., discrimination, chronic stress), language barriers, access to socioeconomic and health-promoting resources (e.g., health insurance), health behaviors (e.g., smoking, alcohol consumption), and coping behaviors (e.g., prayer) may arise from the unique constellations of social positions that individuals occupy [1, 42, 67]. By using an intersectional perspective that first identifies individuals’ positionality and conceptualizes the ways that positionality shapes exposure to unique health risks and access to resources across the life course, scholars are better able to move beyond simply describing group differences to identifying potentially modifiable factors and informing more effective and culturally appropriate interventions to improve outcomes for aging minoritized populations.

**Table 1:** Theoretical frameworks to advance minority aging research

Theory/framework	Overview	Recommended reading(s)
Intersectionality theory	Highlights how social structural dynamics and systems of oppression, such as racism, sexism, capitalism, and heteropatriarchy, intersect and reinforce each other to stratify and dominate minoritized groups by shaping the contexts of their lives	Warner, David F., and Tyson H. Brown. “Understanding how race/ethnicity and gender define age-trajectories of disability: An intersectionality approach.” Social science & medicine 72, no. 8 (2011): 1236–1248 Z. Robinson, “Intersectionality and Gender Theory,” in Handbook of the Sociology of Gender, B. J. Risman, C. Froyum, and W. Scarborough, Eds. Springer International Publishing, 2018
Life course perspective	Examines phenomena at the intersection of social pathways, social change, and developmental trajectories; emphasizes historical time and place and social perspectives across generations, cohorts, or individuals’ lifespans to identify patterns of disease in the context of the social, economic, and cultural environment	Elder, Glen H., Monica Kirkpatrick Johnson, and Robert Crosnoe. “The emergence and development of life course theory.” In Handbook of the life course, pp. 3–19. Springer, Boston, MA, 2003. Harvard
National Institute on Aging health disparities framework	Outlines four key levels of analysis (environmental, sociocultural, behavioral, and biological) that independently and collectively shape health disparities	Hill, Carl V., Eliseo J. Pérez-Stable, Norman A. Anderson, and Marie A. Bernard. “The National Institute on Aging health disparities research framework.” Ethnicity & disease 25, no. 3 (2015): 245
Public health critical race praxis	Provides a semi-structured process for conducting racial equity and methodologically rigorous research and identifies ten principles: race consciousness, primacy of racialization, race as a social construct, ordinariness of racism, structural determinism, social construction of knowledge, critical approaches, intersectionality, disciplinary self-critique, and voice	Ford, Chandra L., and Collins O. Airhihenbuwa. “The public health critical race methodology: praxis for antiracism research.” Social science & medicine 71, no. 8 (2010): 1390–1398
Latina/o critical theory	Complements and supplements critical race theory to highlight the intersectionality of racism, classism, sexism, and nativism and the social and political processes shaping patterns in immigration-related experiences	Bernal, Delgado. “Critical race theory, Latino critical theory, and critical raced-gendered epistemologies.” Education feminism: Classic and contemporary readings 389 (2013)

*Second, intersectionality reveals sources of heterogeneity and distinct health determinants among groups.* Intersectionality theory not only provides theoretical guidance into how structural and individual-level factors create inequities [44], but it also offers insight into methodological strategies for assessing these complex associations. Traditionally, intersectionality studies tended to examine between-group differences, such as disparities between Black and White people, which often masks within-group heterogeneity and group-specific social determinants of health [38]. However, scholars have increasingly challenged this approach, moving away from comparative studies, to focus on within-group studies that examine nuances among specific populations [68, 69]. This within-group analytical approach is a complementary strategy that provides significant value to the field of minority aging because it avoids assumptions of advantaged groups (e.g., Whites, men) as a standard against which marginalized groups must deviate [70]. More specifically, this approach generates opportunities to understand unique determinants of health by looking at specific groups experiencing intersectional disadvantage as opposed to simply comparing them to other groups [71–74]. This strategy also dispels the trend in aging research to treat minoritized populations monolithically by recognizing distinctions that arise *within* these groups due to multiple and oftentimes compounding axes of structural inequities [43]. Moreover, a within-group analytic approach moves beyond the recognition of intersectionality as simply the analysis of interaction terms and allows us to think critically about the substantive meaning and interpretations of multiple exposures faced by populations. Thus, by applying intersectional perspectives and engaging in more within-group studies, minority aging researchers will be better positioned to shed light on the health patterns of minoritized populations.

With respect to data, measurement, and analytic needs, we provide a list of data sources in Table 2 that can be leveraged to conduct minority aging and health research guided by intersectionality. In addition to considering available quantitative datasets, intersectional research on aging minority populations will also benefit from qualitative and mixed-method methodological approaches that give researchers more latitude in constructing their sample and developing measures relevant to specific groups (e.g., Black women, US-born Latino men). Quantitative datasets are limited in scope with regard to including social determinants unique to particular intersectionally disadvantaged groups. Though large data sources include social determinants of health such as discrimination (e.g., available in NSHAP), perceived neighborhood disadvantage (e.g., available in NHATS), and skin tone (e.g., available in HRS), intersectional stressors unique to the experiences of specific multiply marginalized populations are typically not available. An example that may be illustrative here: Black women report being on the receiving end of gendered racial microaggressions and a quantitative 26-item scale was developed to capture these experiences [77]. Though not specifically designed to assess gendered racial microaggressions among older Black women, future qualitative methodologies (e.g., in-depth interviews, focus groups) could be used to reveal the unique ways older Black women experience gendered racism; in turn, such an investigation could catalyze measurement innovation that takes an intersectional approach and identifies how gendered racism manifests in the lives of older Black women.

**Table 2:** Existing data to advance minority aging research

Datasets	Overview	Data details
Health and Retirement Study (HRS) and Sibling Studies	The HRS is a nationally representative biennial panel survey that launched in 1992. Data are collected on a variety of topics, ranging from sociodemographic, behavioral, and health characteristics, including psychosocial and biomarker data starting in 2006. The HRS also provides data for individual countries (e.g., the USA, Mexico, India, Japan) and can also be used for cross-national comparisons	U.S. study: https://hrs.isr.umich.edu/about International studies: https://hrs.isr.umich.edu/about/internationalfamily-studies Juster, F. T., & Suzman, R. (1995). An overview of the Health and Retirement Study. Journal of Human Resources, S7–S56
Hispanic Established Population for the Epidemiological Study of the Elderly (H-EPESE)	The H-EPESE provides estimates of the prevalence of key physical health conditions, mental health conditions, and functional impairments among older Mexican Americans ages 65 and older in Arizona, California, Colorado, New Mexico, and Texas	https://www.icpsr.umich.edu/web/NACDA/series/546
Sacramento Area Latino Study on Aging (Salsa)	The SALSA project tracks the incidence of physical and cognitive impairment, dementia, and cardiovascular diseases among older Latinos in Sacramento, California	https://www.icpsr.umich.edu/web/NACDA/studies/29323
Hispanic Community Health Study/Study of Latinos (HCHS/SOL)	The HCHS/SOL is a prospective cohort study aimed at estimating cardiovascular/pulmonary disease (CVPD) risk factors and disease prevalence and incidence among Latinos	González, Hector M., Wassim Tarraf, Myriam Fornage, Kevin A. González, Albert Chai, Marston Youngblood, Maria de los Angeles Abreu et al. “A research framework for cognitive aging and Alzheimer’s disease among diverse US Latinos: Design and implementation of the Hispanic Community Health Study/Study of Latinos—Investigation of Neurocognitive Aging (SOL-INCA).” Alzheimer’s & Dementia 15, no. 12 (2019): 1624–1632
Rush Memory and Aging Project	The Rush Memory and Aging Project is a longitudinal, epidemiologic clinical-pathologic cohort study in Northeastern Illinois that focuses on chronic conditions, with an emphasis on cognitive and motor function and risk of Alzheimer’s disease	A Bennett, David, Julie A Schneider, Aron S Buchman, Lisa L Barnes, Patricia A Boyle, and Robert S Wilson. “Overview and findings from the rush Memory and Aging Project.” Current Alzheimer Research 9, no. 6 (2012): 646–663
National Social Life, Health, and Aging Project (NSHAP)	NSHAP is a longitudinal, population-based study of health and social factors, aiming to understand the well-being of older, community-dwelling Americans	Waite, L. J., Duvoisin, R., & Kotwal, A. A. (2021). Social Health in the National Social Life, Health, and Aging Project. The Journals of Gerontology: Series B, 76(Supplement_3), S251–S265
National Health & Aging Trends Study (NHATS)	Begun in 2011, the National Health and Aging Trends Study (NHATS) conducts annual in-person interviews with a nationally representative sample of Medicare beneficiaries ages 65 or older	https://www.nhats.org/researcher
Midlife Development in the United States (MIDUS)	First conducted in 1995/1996, this study was conceived by a multidisciplinary team and sought to investigate the role of behavioral, psychological, and social factors in accounting for age-related variations in health and well-being in a national sample of Americans. Two waves of follow up data were collected in 2004–2006 and 2013–2014	https://midus.wisc.edu/index.php
Panel Study of Income Dynamics (PSID)	The PSID is the world’s longest running household panel survey. Starting in 1968, the PSID collects repeated information from individuals and their families covering topics such as employment, income, wealth, expenditures, health, marriage, childbearing, child development, philanthropy, and education	https://psidonline.isr.umich.edu/default.aspx

*Third, and relatedly, an intersectional perspective bolsters scholars’ ability to emphasize health-promoting assets rather than only deficits among aging minoritized groups.* Aging research has traditionally utilized numerous approaches to study racially and ethnically minoritized older adults, including those that accentuate differences or deviations between groups [70]. Nevertheless, inherent in intersectionality theory is the recognition that individuals experience a range of disadvantages and privileges associated with their various social positions [34, 75, 76]. By applying an intersectional lens to minority aging, researchers are better positioned to recognize the ways in which racially minoritized older adults are also advantaged and how those advantages may contribute to improved health. This approach not only centers the experiences of minoritized individuals, but it strengthens our ability to identify effective avenues for intervention and to develop strategies and interventions that strive toward health equity.
